# Early Post-Transplant Changes in Nutritional Status Predict Long-Term Graft Loss and Mortality

**DOI:** 10.3390/jcm15135299

**Published:** 2026-07-07

**Authors:** Taha Enes Cetin, Omer Faruk Akcay, Veysel Baran Tomar, Serhat Haliloglu, Anil Oguzhan Soylu, Kadriye Altok, Yasemin Erten

**Affiliations:** 1 Department of Nephrology, Faculty of Medicine, Gazi University, 06560 Ankara, Türkiye; omerfaruk_akcay@yahoo.com (O.F.A.); veyselbaran.tomar@gmail.com (V.B.T.);; 2 Department of Internal Medicine, Faculty of Medicine, Gazi University, 06560 Ankara, Türkiyeanilsoylu1998@hotmail.com (A.O.S.)

**Keywords:** renal transplantation, nutritional status, prognostic nutritional index (PNI), controlling nutritional status (CONUT), graft loss

## Abstract

**Background:** Nutritional status is an important determinant of outcomes after kidney transplantation, yet the prognostic significance of longitudinal changes in nutritional indices remains insufficiently investigated. We evaluated whether first-year changes in the Prognostic Nutritional Index (PNI) and Controlling Nutritional Status (CONUT) score were associated with long-term adverse outcomes in kidney transplant recipients (KTRs). **Methods:** This retrospective cohort study included 352 adult KTRs with a median follow-up of 11.4 years. PNI and CONUT were calculated before transplantation and at the first post-transplant year. Patients were categorized according to changes in nutritional status as improved/stable or worsened. The primary outcome was a composite of end-stage renal disease (ESRD) or death with a functioning graft. Two separate multivariable logistic regression models were constructed to evaluate the associations of changes in first-year PNI and CONUT with the composite outcome after adjustment for clinically relevant demographic and transplant-specific variables, including first-year rejection episodes, estimated glomerular filtration rate (eGFR), and urine protein-to-creatinine ratio (PCR). Additional analyses were performed separately for graft loss and death with a functioning graft. **Results:** Baseline PNI and CONUT scores were comparable between patients with and without adverse outcomes. Patients who reached the composite outcome had significantly lower first-year eGFR (*p* = 0.001), whereas first-year rejection episodes and urine PCR were similar between the groups. After multivariable adjustment, worsening first-year PNI (OR: 2.02, 95% CI: 1.08–3.77, *p* = 0.028) and worsening first-year CONUT (OR: 2.01, 95% CI: 1.11–3.65, *p* = 0.022) remained independently associated with the composite outcome. Higher first-year eGFR was independently associated with a lower risk of adverse outcomes. Separate analyses of graft loss and death with a functioning graft supported the primary findings, with stronger associations observed for graft-related outcomes. **Conclusions:** Early deterioration in nutritional status, reflected by worsening changes in PNI and CONUT during the first post-transplant year, was independently associated with adverse long-term outcomes in KTRs. Serial assessment of these simple and inexpensive nutritional indices may improve long-term risk stratification and help identify patients who could benefit from closer clinical follow-up and individualized nutritional assessment.

## 1. Introduction

Nutritional status is an important determinant of clinical outcomes and quality of life at all stages of chronic kidney disease (CKD) [1]. Appropriate nutritional interventions can slow disease progression in the early stages of CKD and delay the need for renal replacement therapy [2]. CKD patients are at high risk of nutritional disorders, including protein–energy wasting and electrolyte imbalances, mainly due to inadequate intake, chronic inflammation, and the accumulation of uremic toxins [3,4]. These disturbances contribute to losses in muscle and fat mass, increased frailty, greater susceptibility to infection, higher hospitalization rates, and ultimately increased mortality [5,6].

Composite nutritional assessment tools such as the Prognostic Nutritional Index (PNI) and the Controlling Nutritional Status (CONUT) score are widely used in clinical practice to evaluate both nutritional and immune status. Both indices are derived from routinely measured laboratory parameters, making them inexpensive, readily available, and easily reproducible during standard clinical follow-up. While both incorporate serum albumin and lymphocyte count, CONUT additionally includes total cholesterol, providing a broader assessment of protein–energy balance [7,8]. Across studies involving malignancies, postoperative settings, heart failure, CKD, and end-stage renal disease (ESRD), low PNI and high CONUT scores have consistently been associated with adverse clinical outcomes, including increased mortality, infection, cardiovascular events, prolonged hospitalization, postoperative complications, and poor survival [9,10,11,12,13,14,15]. More recently, these indices have also demonstrated prognostic value in kidney transplant recipients (KTRs), supporting their use as practical and readily applicable tools for post-transplant nutritional risk assessment [16,17].

Kidney transplantation (KT) substantially improves survival and quality of life in patients with ESRD [18]. However, nutritional status remains an underrecognized component of pretransplant evaluation despite its clear influence on perioperative risk and long-term outcomes [19]. Malnutrition and low body mass index have been linked to higher rates of surgical complications, infections, prolonged hospitalization, and mortality following KT [20,21]. 

Notably, malnutrition is common among KTRs and has been strongly associated with adverse post-transplant outcomes [22].

While baseline nutritional status is known to affect outcomes in KTRs, less is known about how post-transplant nutritional changes influence long-term prognosis. The first year after transplantation is a critical period marked by immunosuppression, inflammation, metabolic shifts, and recovery of renal function [23]. These factors may dynamically impact nutritional status, yet the prognostic value of such changes has not been clearly established. Most existing studies have relied on static, cross-sectional assessments, which may overlook meaningful fluctuations during this vulnerable phase. Therefore, this study aimed to evaluate whether changes in PNI and CONUT scores during the first year predict long-term graft and patient outcomes in KTRs. By focusing on early changes in nutritional status rather than baseline values alone, this research seeks to provide clinically actionable insights into early nutritional risk stratification after transplantation.

## 2. Methods

### 2.1. Study Design and Population

This retrospective cohort study included adult KTRs with complete baseline and first-year laboratory data required to calculate the PNI and CONUT scores. Because the primary exposure variables (ΔPNI and ΔCONUT) were defined as the changes between baseline and the first post-transplant year, only patients with available nutritional assessments at both time points were eligible. Consequently, patients who died, experienced graft loss, or lacked follow-up before the first post-transplant year were excluded, resulting in a 1-year landmark cohort. Accordingly, all exposure variables were defined before long-term outcome assessment, and the analyses were performed within this predefined landmark cohort. A complete-case approach was used for all analyses; therefore, no multiple imputation or other missing-data methods were required. Outcome data for graft loss and mortality were available for all patients included in the analytical cohort.

Induction therapy was based on immunological risk and included basiliximab, daclizumab, or anti-thymocyte globulin (ATG), while low-risk patients typically received no induction. Maintenance immunosuppression included a calcineurin inhibitor (tacrolimus or cyclosporine), an antimetabolite (mycophenolate mofetil or azathioprine), and low-dose corticosteroids. Mammalian target of rapamycin (mTOR) inhibitors were used in cases of intolerance to calcineurin inhibitors or antimetabolites. The study was conducted in accordance with the ethical principles of the Declaration of Helsinki and was approved by the Ethics Committee of Gazi University (Research Code No: 2025-1370; Approval Date: 29 July 2025).

### 2.2. Data Collection

Medical records were systematically reviewed to obtain demographic, clinical, and transplant-related information for all KTRs included in the study. Demographic data consisted of age, sex, comorbidities [diabetes mellitus (DM), hypertension (HT), and coronary artery disease (CAD)], smoking status, and the primary cause of ESRD. Pre-transplant clinical variables included dialysis duration, dialysis modality, and baseline laboratory measurements relevant to nutritional assessment, including serum albumin, total lymphocyte count, and total cholesterol levels. Transplant-related variables included donor type (living vs. deceased), induction therapy (none, IL-2 receptor antagonist, or ATG), the number of Human Leukocyte Antigen (HLA) mismatches, preemptive transplantation status, New-Onset Diabetes After Transplantation (NODAT), and the presence of delayed graft function, defined as the requirement for dialysis within the first post-transplant week. In addition, first-year transplant-specific variables, including biopsy-proven acute rejection episodes, estimated glomerular filtration rate (eGFR), and the urine protein-to-creatinine ratio (PCR), were collected and evaluated as potential prognostic variables.

### 2.3. Nutritional Indices

PNI was calculated using the validated formula [8].

PNI = (10 × serum albumin [g/dL]) + (0.005 × total lymphocyte count [/mm^3^])

The CONUT score incorporates serum albumin, lymphocyte count, and total cholesterol, with points assigned based on defined thresholds to yield a total score from 0 to 12; higher scores indicate poorer nutritional status [7].

### 2.4. Calculation and Classification of ΔPNI and ΔCONUT

To quantify changes in nutritional status during the first post-transplant year, delta values were calculated as follows:

- ΔPNI = PNI score first-year − PNI score baseline
- ΔCONUT = CONUT score first-year − CONUT score baseline

A positive ΔPNI was interpreted as improved nutritional status, whereas a negative ΔPNI indicated worsening [24]. Accordingly, patients were classified as follows:

- Improved/Stable PNI: ΔPNI ≥ 0;
- Worsened PNI: ΔPNI < 0.

Because higher CONUT scores represent worsening nutritional status [25], patients were classified as follows:

- Improved/Stable CONUT: ΔCONUT ≤ 0;
- Worsened CONUT: ΔCONUT > 0.

### 2.5. Outcome Definition

The primary outcome was a composite of ESRD or death with a functioning graft during follow-up. Patients were divided into two groups based on the presence or absence of this composite endpoint. Additional multivariable analyses were performed separately for graft loss and death with a functioning graft to evaluate the individual components of the composite endpoint.

### 2.6. Statistical Analysis

The normality of data distribution was assessed using the Kolmogorov–Smirnov test. Continuous variables were expressed as mean ± standard deviation or median (interquartile range), and group comparisons were performed using Student’s *t*-test or Mann–Whitney U test, as appropriate. Paired comparisons of baseline and first-year nutritional indices (PNI and CONUT scores) were performed using the Wilcoxon signed-rank test because of the non-normal distribution of the data. Candidate covariates for the multivariable models were selected based on their clinical relevance and the results of the univariate analyses. To reduce the risk of model overfitting given the limited number of outcome events, variables with a *p* value <0.10 in the corresponding univariate analysis were considered for inclusion in the multivariable models. Clinically relevant transplant-specific variables, including first-year rejection episodes, eGFR, and urine PCR, were additionally evaluated as potential confounders. Two separate multivariable logistic regression models were constructed to evaluate the association between changes in nutritional status and the composite outcome of ESRD or death. In Model 1, the primary independent variable was worsened ΔPNI status (vs. improved/stable), whereas in Model 2, it was worsened ΔCONUT status (vs. improved/stable). Because PNI and CONUT share common components (serum albumin and lymphocyte count), they were analyzed in separate models to avoid multicollinearity. Multicollinearity was assessed before model construction, and no evidence of significant collinearity was identified among the included covariates. Additional multivariable logistic regression analyses were also performed separately for graft loss and death with a functioning graft using the same predefined modeling strategy. Results are presented as odds ratios (ORs) with 95% confidence intervals (CIs). A two-sided *p* value < 0.05 was considered statistically significant. Statistical analyses were performed using SPSS version 23.0 (IBM Corp., Armonk, NY, USA).

## 3. Results

### 3.1. Baseline Demographic and Clinical Characteristics

A total of 352 KTRs were included in the study, of whom 95 (27.0%) developed the composite outcome of ESRD or death during follow-up (Table 1). The median age at transplantation was slightly higher in patients with adverse outcomes than those without (37.5 ± 16.1 vs. 33.9 ± 13.2 years, *p* = 0.058). The sex distribution was similar between the groups (female: 62.1% vs. 62.6%, *p* = 0.926). Baseline comorbidities revealed a higher frequency of HT among patients with ESRD or death (78.9% vs. 66.1%, *p* = 0.020), while CAD showed only a borderline association with adverse outcomes (15.8% vs. 8.6%, *p* = 0.050). No significant differences were observed in the prevalence of DM, smoking status, NODAT, or dialysis duration. Baseline laboratory parameters, including albumin, hemoglobin, lymphocyte count, platelet count, and lipid profile, were comparable between the groups.

Pretransplant nutritional indices were similar between the groups, with no significant differences in baseline PNI (*p* = 0.539) or CONUT score (*p* = 0.368). The median follow-up duration was 11.4 years (IQR 6.0–16.0), also without a significant between-group difference. At the first post-transplant year, patients who reached the composite outcome had significantly lower eGFR than those without an event (66.7 [50.8–83.0] vs. 77.3 [63.5–98.5] mL/min/1.73 m^2^, *p* = 0.001), whereas first-year urine PCR and rejection episodes were comparable between the groups (*p* = 0.615 and *p* = 0.558, respectively).

### 3.2. Nutritional Indices and Temporal Changes

**Figure 1:**
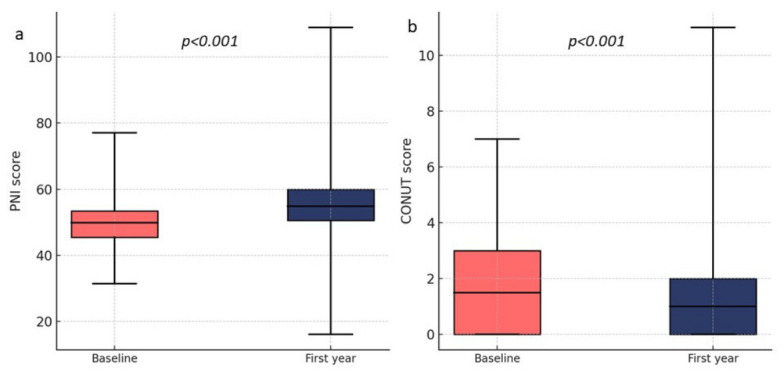
(**a**,**b**) Boxplots showing baseline and first-year PNI and CONUT scores, illustrating changes in nutritional status during the first post-transplant year.

Figure 1a,b display the distribution of PNI and CONUT scores at baseline and one year after transplantation. Median PNI increased from 49.8 (IQR 45.4–53.5) to 54.9 (IQR 50.5–60.0), while the median CONUT score decreased from 1.5 (IQR 0–3.0) to 1.0 (IQR 0–2.0), indicating significant overall improvement in nutritional parameters (both *p* < 0.001).

**Figure 2:**
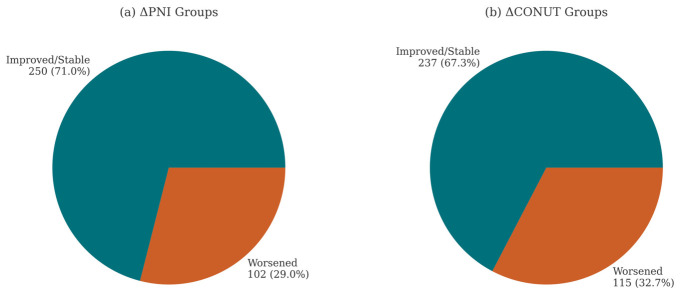
(**a**,**b**) Distribution of patients classified as improved/stable or worsened according to ΔPNI and ΔCONUT during the first post-transplant year.

**Figure 3:**
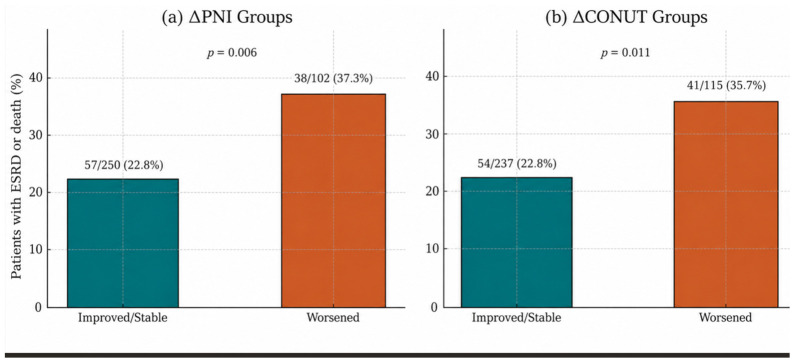
(**a**,**b**) Proportion of patients reaching the composite outcome (ESRD or Death) according to first-year ΔPNI and ΔCONUT changes.

Based on changes in nutritional indices, 71.0% of patients (*n*= 250) were classified as having improved or stable PNI, while 29.0% (*n* = 102) showed a decline. For CONUT, 67.3% (*n* = 237) had improved or stable scores, and 32.7% (*n* = 115) experienced deterioration (Figure 2a,b). The incidence of ESRD or death was 22.8% in patients with improved/stable PNI compared to 37.3% in those with worsened PNI (*p* = 0.006). For CONUT, these rates were 22.8% and 35.7%, respectively (*p* = 0.011) (Figure 3a,b). The incidence of the composite outcome was significantly higher among patients with worsened PNI and CONUT status.

### 3.3. Regression Analyses

Univariate logistic regression identified several variables associated with the composite endpoint (Table 2). Older age was associated with an increased risk of ESRD or death (OR: 1.02, 95% CI 1.00–1.04, *p* = 0.038). HT also significantly predicted adverse outcomes (OR: 1.92, 95% CI 1.10–3.35, *p* = 0.022). Worsening nutritional parameters demonstrated strong associations: worsened first-year PNI status (OR: 2.01, 95% CI 1.22–3.31, *p* = 0.006) and worsened CONUT status (OR: 1.88, 95% CI 1.15–3.06, *p* = 0.011). CAD demonstrated a borderline association with the composite outcome (OR: 2.00, 95% CI 0.99–4.05, *p* = 0.053), while delayed graft function (OR: 1.82, 95% CI 0.89–3.73, *p* = 0.100) and NODAT (OR: 1.69, 95% CI 0.93–3.07, *p* = 0.086) showed trends toward significance.

Variables meeting the predefined selection criteria were entered into two separate multivariable logistic regression models. After adjustment for transplant-specific variables, including first-year rejection episodes, first-year eGFR, and first-year urine PCR, worsened ΔPNI (OR: 2.02, 95% CI 1.08–3.77, *p* = 0.028) in Model 1 and worsened ΔCONUT (OR: 2.01, 95% CI 1.11–3.65, *p* = 0.022) in Model 2 remained independently associated with the composite outcome. In both models, higher first-year eGFR was independently associated with a lower risk of ESRD or death (Table 3).

To further evaluate the individual components of the composite endpoint, additional multivariable logistic regression analyses were performed separately for graft loss and death with a functioning graft (Supplementary Tables S1 and S2). In the graft loss analysis, worsening ΔPNI remained independently associated with graft loss, whereas worsening ΔCONUT showed a similar direction of association. In contrast, neither nutritional index remained independently associated with death with a functioning graft after adjustment for transplant-specific variables.

## 4. Discussion

Our findings suggest that early post-transplant changes in nutritional status are associated with long-term outcomes after KT. Although overall PNI and CONUT scores generally improved during the first post-transplant year, nearly one-third of patients showed deterioration in these indices. Notably, despite having similar baseline nutritional profiles, those who experienced a decline in PNI or an increase in CONUT were significantly more likely to develop graft loss or death. As conventional clinical parameters may not fully capture early deterioration, monitoring first-year changes in nutritional status offers a practical, clinically meaningful strategy to identify patients who may require closer follow-up and timely supportive interventions.

Early post-transplant nutritional deterioration likely reflects a convergence of biological mechanisms that heighten long-term risk. The first post-transplant year is characterized by immune modulation, variable inflammatory burden, shifts in protein metabolism, and fluctuations in lymphocyte counts due to immunosuppressive therapy [26]. Declines in albumin, lymphocytes, or cholesterol, the components of both PNI and CONUT, may therefore signal systemic inflammation, impaired immune competence, increased susceptibility to infection, and reduced metabolic reserve [27]. These mechanisms provide a plausible biological explanation for the observed association between worsening nutritional status and major adverse outcomes, including ESRD and mortality.

Overall, nutritional status improved during the first post-transplant year, as reflected by rising PNI values and declining CONUT scores. This pattern is consistent with the well-recognized metabolic and inflammatory benefits of successful KT [28]. Restoration of renal function reduces uremic toxin load, improves appetite and nutrient intake, corrects metabolic acidosis, and enhances protein synthesis, thereby promoting recovery of nutritional reserves [29,30]. However, a significant proportion of patients exhibited worsening changes in nutritional status, which were associated with adverse long-term outcomes. This finding suggests that patients who fail to demonstrate the expected nutritional recovery during the first post-transplant year may represent a subgroup at higher risk for adverse long-term outcomes.

Previous studies have consistently demonstrated that higher PNI values are associated with better survival among KTRs [17], reflecting the importance of preserved protein reserves and immune competence in long-term outcomes. Likewise, the CONUT score has shown prognostic value in other nephrology populations, particularly in predicting early mortality among hemodialysis patients [31,32]. Despite growing clinical interest, evidence regarding the longitudinal evolution of PNI and CONUT after KT remains limited, as most previous studies have relied on cross-sectional assessments that provide only a static snapshot of nutritional status [16,33]. To the best of our knowledge, no previous study has evaluated longitudinal changes in CONUT specifically within the transplant population. By examining both baseline values and first-year changes, our study addresses this important gap.

Importantly, PNI and CONUT should not be interpreted as pure nutritional markers. Because both indices incorporate laboratory parameters influenced by inflammation, infection, immunosuppressive therapy, fluid status, and graft function, worsening scores may reflect underlying systemic illness and allograft-related complications in addition to nutritional deterioration [26,27,34]. Rather than representing a limitation, this broader biological sensitivity may enhance their clinical utility by allowing them to serve as simple and readily available indicators of overall patient vulnerability. Future prospective studies incorporating comprehensive nutritional assessments, inflammatory biomarkers, body composition measurements, rejection episodes, proteinuria, and longitudinal graft function are needed to better clarify the mechanisms underlying these associations and to determine whether targeted interventions can improve long-term outcomes.

A major strength of this study is the long-term follow-up of a large, well-characterized cohort of KTRs, enabling robust evaluation of clinically meaningful long-term outcomes. By assessing dynamic changes in both PNI and CONUT during the first post-transplant year, we demonstrated that these early nutritional changes provide clinically relevant prognostic information beyond baseline measurements. Additional analyses evaluating graft loss and mortality separately further supported the primary findings and suggested that the prognostic value of early changes in nutritional status may be driven predominantly by graft-related outcomes rather than mortality alone.

## 5. Limitations

This study has several limitations. As a single-center study, the generalizability of our findings to broader transplant populations may be restricted. Second, because this study was designed as a one-year landmark cohort, only patients with available nutritional assessments at baseline and during the first post-transplant year were included. Therefore, reverse causality cannot be completely excluded, and competing-risk regression and landmark sensitivity analyses were not performed. Biochemical indices such as PNI and CONUT can be influenced by hydration status, inflammation, infections, surgery-related complications, and immunosuppressive therapy. As no clinically validated cutoff or minimal clinically important difference has been established for ΔPNI or ΔCONUT in KTRs, alternative classification strategies and threshold values should be evaluated in future studies. Third, the absence of more comprehensive nutritional assessments, such as body composition, dietary intake, or physical performance measures, limited the characterization of nutritional status. Although first-year transplant-specific variables, including rejection episodes, eGFR, and urine PCR, were included in the analyses, other clinically relevant factors, such as longitudinal graft function and changes in immunosuppressive therapy, were not consistently available. In addition, because of the retrospective design and long follow-up period, some longitudinal laboratory measurements were unavailable because follow-up was occasionally performed at other institutions, although complete outcome data were available for all patients included in the analytical cohort. Finally, detailed measures of pre-transplant dialysis adequacy were unavailable, and although the number of covariates was carefully restricted, the relatively limited number of death events may have affected the stability of the mortality models.

## 6. Conclusions

In conclusion, deterioration in nutritional status during the first post-transplant year, reflected by worsening changes in PNI and CONUT, was independently associated with adverse long-term outcomes in KTRs. These findings suggest that longitudinal assessment of nutritional indices may provide additional prognostic information beyond baseline measurements. Given that PNI and CONUT are inexpensive, readily available, and routinely obtainable in clinical practice, serial evaluation of these indices may help identify patients who could benefit from closer clinical follow-up and individualized nutritional assessment. Further prospective multicenter studies are needed to validate these findings and determine whether nutritional management strategies can improve long-term transplant outcomes.

## Figures and Tables

**Table 1:** Baseline Demographic, Clinical, and Nutritional Characteristics Stratified by Adverse Outcome (ESRD or Death).

	Total *n* 352	No *n*: 257 (73.0%)	ESRD or Death *n*: 95 (27.0%)	*p* Value *
Age at transplantation (years)	34.9 ± 14.1	33.9 ± 13.2	37.5 ± 16.1	0.058
Female sex, *n* (%)	220 (62.5)	161 (62.6)	59 (62.1)	0.926
Diabetes Mellitus, *n* (%)	36 (10.2)	26 (10.1)	10 (10.5)	0.910
Hypertension, *n* (%)	245 (69.6)	170 (66.1)	75 (78.9)	**0.020**
CAD, *n* (%)	37 (10.5)	22 (8.6)	15 (15.8)	**0.050**
NODAT, *n* (%)	58 (16.5)	37 (14.4)	21 (22.1)	0.084
Smoking, *n* (%)	89 (25.3)	60 (23.3)	29 (30.5)	0.169
Cause of ESRD, *n* (%) Hypertension Diabetes mellitus Glomerulonephritis VUR ADPKD Others Unknown	32 (9.1) 22 (6.3) 79 (22.4) 28 (8.0) 24 (6.8) 88 (25.0) 79 (22.4)	25 (9.7) 14 (5.4) 62 (24.1) 19 (7.9) 18 (7.0) 64 (24.9) 55 (21.4)	8 (8.4) 7 (7.4) 17 (17.9) 9 (9.5) 6 (6.3) 24 (25.3) 24 (25.3)	0.745
Dialysis duration (years)	5.04 ± 4.68	4.8 ± 4.6	5.6 ± 4.9	0.274
HLA mismatch, (IQR)	3 (2–3)	3 (2–3)	3 (2–3)	0.446
Preemptive transplantation, *n* (%)	95 (27.0)	71 (27.6)	24 (25.3)	0.657
Donor type, *n* (%) Deceased	81 (23.0)	54 (21.0)	27 (28.4)	0.143
Induction, *n* (%) None IL-2 receptor antagonist ATG	108 (30.7) 101 (28.7) 143 (40.6)	83 (32.3) 70 (27.2) 104 (40.5)	25 (26.3) 31 (32.6) 39 (41.1)	0.468
Delayed graft function, *n* (%)	36 (10.3)	22 (8.7)	14 (14.7)	0.097
Pretransplant Laboratory Values Albumin (g/dL) Hemoglobin (g/dL) Lymphocyte count (/mm^3^) Platelet count (×10^3^/mm^3^) Total cholesterol (mg/dL)	4.1 ± 0.5 10.6 ± 1.9 1715 ± 784 214 ± 73 181 ± 47	4.1 ± 0.5 10.6 ± 1.9 1725 ± 816 216 ± 72 180 ± 48	4.1 ± 0.5 10.5 ± 2.0 1690 ± 694 208 ± 77 183 ± 43	0.411 0.865 0.715 0.351 0.590
PNI score, (IQR)	49.8 (45.4–53.5)	49.6 (45.4–53.0)	50.2 (45.3–54.9)	0.539
CONUT score, (IQR)	1.5 (0–3.0)	1 (0–3)	2 (0–2)	0.368
First-year eGFR (mL/min/1.73 m^2^)	74.6 (60.7–96.0)	77.3 (63.5–98.5)	66.7 (50.8–83.0)	**0.001**
First-year urine PCR (mg/g)	200 (115–315)	200 (115–310)	200 (130–320)	0.615
First-year rejection episodes, *n* (%)	25 (7.1)	17 (6.6)	8 (8.4)	0.558
Follow-up duration (years)	11.4 ± 6.0	11.1 ± 5.8	12.2 ± 6.4	0.138

ADPKD: Autosomal Dominant Polycystic Kidney Disease, ATG: Anti-thymocyte globulin, CAD: Coronary artery disease, CONUT: Controlling Nutritional Status score, ESRD: End-stage renal disease, IL-2: Interleukin-2, NODAT: New-onset diabetes after transplantation, PCR: Protein-to-creatinine ratio, PNI: Prognostic Nutritional Index, VUR: Vesicoureteral Reflux. Bold values indicate statistical significance (* *p* < 0.05).

**Table 2:** Univariate logistic regression analysis for predictors of adverse outcomes (ESRD or Death).

	OR	95% CI	*p* Value
Sex (Male)	0.98	0.60–1.59	0.926
Age at transplantation	1.02	1.00–1.04	**0.038**
Induction IL-2 receptor antagonist ATG	1.25 1.47	0.70–2.22 0.80–2.72	0.458 0.220
NODAT	1.69	0.93–3.07	**0.086**
Donor type (Deceased)	1.49	0.87–2.56	0.144
HLA mismatch	1.07	0.89–1.29	0.472
Preemptive transplantation	0.89	0.52–1.52	0.658
Delayed graft function	1.82	0.89–3.73	**0.100**
Hypertension	1.92	1.10–3.35	**0.022**
Diabetes mellitus	1.05	0.48–2.26	0.910
CAD	2.00	0.99–4.05	**0.053**
Smoking	1.44	0.86–2.44	0.170
First-year eGFR	0.98	0.97–0.99	**0.001**
First-year urine PCR	1.00	1.00–1.00	0.739
First-year rejection episodes	0.86	0.25–2.97	0.805
First-year PNI status (worsened)	2.01	1.22–3.31	**0.006**
First-year CONUT status (worsened)	1.88	1.15–3.06	**0.011**

ATG: Anti-Thymocyte Globulin, CAD: Coronary Artery Disease, CI: Confidence Interval, CONUT: Controlling Nutritional Status score, HLA: Human Leukocyte Antigen, IL-2: Interleukin-2, NODAT: New-Onset Diabetes After Transplantation, OR: Odds Ratio, PCR: Protein-to-creatinine ratio, PNI: Prognostic Nutritional Index. Bold *p* values indicate variables selected for inclusion in the multivariable analysis (*p* ≤ 0.10).

**Table 3:** Multivariable logistic regression models evaluating the association of first-year changes in nutritional status with the composite outcome (ESRD or Death).

Variable	Model 1 (ΔPNI) OR (95% CI)	*p* Value	Model 2 (ΔCONUT) OR (95% CI)	*p* Value
Age at transplantation	1.00 (0.97–1.02)	0.780	1.00 (0.98–1.02)	0.955
Delayed graft function	1.19 (0.51–2.78)	0.695	1.47 (0.63–3.39)	0.372
NODAT	1.42 (0.70–2.85)	0.330	1.48 (0.73–2.99)	0.274
Hypertension	1.36 (0.69–2.67)	0.379	1.31 (0.66–2.58)	0.438
CAD	1.41 (0.55–3.60)	0.472	1.53 (0.60–3.91)	0.377
First-year eGFR	0.98 (0.97–0.99)	0.005	0.98 (0.97–0.99)	**0.007**
First-year urine PCR	1.00 (1.00–1.00)	0.952	1.00 (1.00–1.00)	0.913
First-year rejection episodes	0.99 (0.35–2.78)	0.982	1.11 (0.39–3.19)	0.842
Worsened ΔPNI	2.02 (1.08–3.77)	0.028	—	—
Worsened ΔCONUT	—	—	2.01 (1.11–3.65)	**0.022**

CAD: Coronary Artery Disease, CI: Confidence Interval, CONUT: Controlling Nutritional Status score, eGFR: estimated glomerular filtration rate, NODAT: New-Onset Diabetes After Transplantation, OR: Odds Ratio, PCR: Protein-to-creatinine ratio, PNI: Prognostic Nutritional Index. Bold values indicate statistical significance (*p* < 0.05).

## Data Availability

Data supporting this study are available from the corresponding author on reasonable request, subject to ethics approval and data protection requirements.

## Supplementary Materials

The following supporting information can be downloaded at https://www.mdpi.com/article/10.3390/jcm15135299/s1, Table S1: Multivariable logistic regression models evaluating factors associated with graft loss (57 events); Table S2: Multivariable logistic regression models evaluating factors associated with death with a functioning graft (38 events).
