# Supplementary material for: Early Post-Transplant Changes in Nutritional Status Predict Long-Term Graft Loss and Mortality
Source: J Clin Med. 2026 Jul 7;15(13):5299. doi: 10.3390/jcm15135299 (PMC13363453; doi:10.3390/jcm15135299)
Supplement: Supplementary file 1 [file jcm-15-05299-s001.zip › jcm-4379169-supplementary.pdf]

Supplementary Table S1. Multivariable logistic regression models evaluating factors associated with graft loss (57 events)

Variable	Model 1 (ΔPNI) OR (95% CI)	*p* value	Model 2 (ΔCONUT) OR (95% CI)	*p* value
**Hypertension**	1.98 (0.86–4.54)	0.106	2.01 (0.88–4.60)	0.097
**Delayed graft function**	1.88 (0.74–4.74)	0.183	1.82 (0.72–4.63)	0.206
**Age at transplantation**	0.95 (0.92–0.98)	**<0.001**	0.95 (0.92–0.98)	**<0.001**
**First-year eGFR**	0.97 (0.96–0.99)	**<0.001**	0.97 (0.96–0.99)	**<0.001**
**First-year urine PCR**	1.00 (1.00–1.00)	0.764	1.00 (1.00–1.00)	0.896
**First-year rejection episodes**	0.71 (0.22–2.32)	0.573	0.67 (0.20–2.18)	0.502
**Worsened ΔPNI**	2.13 (1.03–4.39)	**0.041**	—	—
**Worsened ΔCONUT**	—	—	2.01 (1.11–3.65)	**0.022**

CI: Confidence Interval, CONUT: Controllng Nutritional Status score, eGFR: Estimated glomerular filtration rate, OR: Odds Ratio, PCR: protein-to-creatinine ratio, PNI: Prognostic Nutritional Index.

Supplementary Table S2. Multivariable logistic regression models evaluating factors associated with death with a functioning graft (38 events)

Variable	Model 1 (ΔPNI) OR (95% CI)	*p* value	Model 2 (ΔCONUT) OR (95% CI)	*p* value
**Age at transplantation**	1.09 (1.05–1.13)	**<0.001**	1.09 (1.05–1.13)	**<0.001**
**First-year eGFR**	1.01 (0.99–1.03)	0.594	1.01 (0.98–1.03)	0.651
**First-year urine PCR**	1.00 (0.99–1.00)	0.767	1.00 (0.99–1.00)	0.772
**First-year rejection episodes**	1.01 (0.18–5.72)	0.989	1.01 (0.18–5.72)	0.988
**Worsened ΔPNI**	1.23 (0.49–3.08)	0.659	—	—
**Worsened ΔCONUT**	—	—	1.37 (0.56–3.33)	0.495

CI: Confidence Interval, CONUT: Controlling Nutritional Status score, eGFR: Estimated glomerular filtration rate, OR: Odds Ratio, PNI: Prognostic Nutritional Index, PCR: Protein-to-creatinine ratio.

* Variables included in each multivariable model were selected according to the predefined modeling strategy described in the Methods section. Clinically relevant transplant-specific variables (first-year rejection episodes, first-year eGFR, and first-year urine protein-to-creatinine ratio) were included in all models irrespective of their univariate significance, whereas the remaining covariates were selected according to the results of the corresponding univariate analyses. Consequently, the covariates included in the multivariable models differed according to the outcome being analyzed.
